# Gut Bless Your Pain—Roles of the Gut Microbiota, Sleep, and Melatonin in Chronic Orofacial Pain and Depression

**DOI:** 10.3390/biomedicines10071528

**Published:** 2022-06-28

**Authors:** Łukasz Lassmann, Matteo Pollis, Agata Żółtowska, Daniele Manfredini

**Affiliations:** 1 Dental Sense Medicover, 80-283 Gdańsk, Poland; 2 Department of Medical Biotechnology, School of Dentistry, University of Siena, 53100 Siena, Italy; matteo.pollis@gmail.com (M.P.); daniele.manfredini75@gmail.com (D.M.); 3 Department of Conservative Dentistry, Faculty of Medicine, Medical University of Gdańsk, 80-210 Gdańsk, Poland; agata.zoltowska@gumed.edu.pl

**Keywords:** gut microbiota, temporomandibular disorders, orofacial pain, bruxism, melatonin, sleep, depression, gastroesophageal reflux disease, irritable bowel syndrome, inflammation

## Abstract

**Background.** Increased attention has been paid to the gut–brain axis recently, but little is known so far regarding how this translates into pain susceptibility. **Aim.** The aim of this review is to determine whether gastroenterological disorders and sleep disorders (directly or indirectly) contribute to an increased susceptibility to depression and chronic orofacial pain. **Method.** A search was performed in the U.S. National Library of Medicine (PubMed) database in order to find studies published before 19 December 2021. We used the following terms: gut microbiome, OR sleep quality, OR melatonin, OR GERD, OR IBS, AND: depression OR chronic pain, in different configurations. Only papers in English were selected. Given the large number of papers retrieved in the search, their findings were described and organized narratively. **Results.** A link exists between sleep disorders and gastroenterological disorders, which, by adversely affecting the psyche and increasing inflammation, disturb the metabolism of tryptophan and cause excessive microglial activation, leading to increased susceptibility to pain sensation and depression. **Conclusions.** Pain therapists should pay close attention to sleep and gastrointestinal disorders in patients with chronic pain and depression.

## 1. Introduction

Among the orofacial pain conditions, the most prominent are the temporomandibular disorders (TMDs) [1]. These disorders affect about 10–15% of the population at a clinically relevant level [2], with 90% of cases reporting pain in the masticatory muscles and tenderness to palpation [3,4].

TMDs are often managed in the dental office; however, they are complex medical conditions, being unrelated to the features of dental occlusion and requiring an interdisciplinary approach. A current gap in TMD practice is the difficulty in training clinicians with respect to the need for evaluation of possible vulnerabilities that are not directly associated with the masticatory organ [5].

Recent suggestions have highlighted the importance of multidisciplinary rehabilitation in the management of chronic pain [6] and gastrointestinal diseases, including gastroesophageal reflux disease (GERD) and irritable bowel syndrome (IBS). It turns out that both GERD and IBS are separately associated with an almost three times higher risk of TMD [7], and the interplay between these diseases is at least partially connected with GERD anti-acidic treatment, which significantly disrupts the gut microbiota—the population of micro-organisms that colonizes the intestines. These micro-organisms produce metabolites, such as serotonin, dopamine, gamma-aminobutyric acid (GABA), and short-chain fatty acids (SCFA), which affect the activity of the central nervous system (CNS) [8,9,10]. This action may modulate many types of chronic pain, including visceral, inflammatory, headache, and neuropathic pain [11]. In addition, some experimental studies have shown that sleep deprivation increases pain sensitivity and leads to hyperalgesia [12,13]. Studies have also confirmed the complete normalization of pain sensation after so-called “recovery sleep” [14], which seems to be important in terms of the possible TMD–sleep connection [15,16,17,18,19]. Moreover, sleep disorders increase susceptibility to stress [20] and have a negative effect on the intestines [21], which, in turn, enhances the stress response [22]. For these reasons, it seems probable that an interaction between sleep disturbances, gut microbiota alteration, and the chronicization of pain may exist. A considerable amount of evidence has contributed to extending the knowledge around this interesting phenomena in recent years; however, the complexity of the interactions and the multiple pathophysiological processes involved make it difficult to understand. Therefore, the aim of this review is to explore the complex picture of individual vulnerability, genetics, and developmental disorders, which is likely to also include factors related to sleep and the gastrointestinal system. 

To the best of our knowledge, this is the first paper presenting possible TMD–pain models involving the inclusion and discussion of quality of sleep, psychological factors (i.e., somatization, depression, anxiety, and PTSD), and gastrointestinal factors (i.e., GERD, IBS, and gut microbiome). Furthermore, several substances that appear to have a positive effect on each of these risk factors are presented. We devote an entire section to the most important of these substances—melatonin. Finally, a possible sequence of events leading to chronic orofacial pain will be presented, with a possible answer to the question of why women suffer more.

## 2. Search Strategy and Selection Criteria 

A search was performed in the U.S. National Library of Medicine (PubMed) database, in order to find studies published before 19 December 2021. We used the following terms to identify possible chronic orofacial pain contributors and their relationships: gut microbiome, OR sleep quality, OR melatonin, OR GERD, OR IBS. These were combined with terms to determine the outcomes of interest: depression OR chronic pain. Inclusion criteria were limited to: (1) relevant papers describing the relationship between at least two of the terms mentioned; (2) papers with statistically significant outcomes; (3) papers focused on the etiology of chronic pain and depression; and (4) studies written in English. The primary author (L.L) independently reviewed the titles and abstracts of all articles, followed by a full-text eligibility check. Any studies not fulfilling the inclusion criteria were excluded from further evaluation, resulting in the eventual qualification and inclusion of 142 out of 1180 papers. Given the large number of papers retrieved in the search, their findings are described and organized narratively in the following. 

## 3. Sleep–Psycho–Pain Axis

Modern sleep research has shown that sleep disruption is linked to the development and progression of anxiety disorders [23,24,25]—the most common mental illness worldwide at present [26].

Interestingly, it has been reported that REM sleep acts as a potential night-time psychotherapist for anxiety and stress [20]. Neuroimaging studies have revealed significant activity increases during REM sleep in emotion-related regions [27,28,29], where these changes are regulated by striking neurochemical alterations [30,31,32]. Perhaps most remarkable is a substantial reduction in the level of noradrenaline during REM sleep [31,33,34,35,36], the lowest at any time during the 24 h period. In this calm, noradrenalin-free environment, REM sleep involves very emotional and often very aggressive dreams [37,38,39], serving to transform emotional memories into memories that are no longer emotional. Therefore, after a good night of sleep, something that previously stressed us may no longer arouse our fears [36]. It is this mechanism that fails in the case of PTSD, where the brain cannot clear itself from excessive amounts of noradrenaline [37].

Non-rapid eye movement slow-wave oscillations (NREM-SW) also offer an ameliorating, anxiolytic benefit [38], but the role of NREM sleep can be considered separate from that of REM sleep in the regulation of emotions [39]. Specifically, (NREM-associated) anxiety is a state that operates across a time-frame of hours, while (REM-associated) emotional reactivity is considered to be a short-term, acute process that begins and ends within a time frame of milliseconds to minutes [40].

Studies in animals [41] and humans have established that total [42], partial [43], and selective sleep deprivation [44,45] can lead to increased pain. It is particularly the lack of the deep phase of non-REM sleep that increases pain sensitivity [46]. One of the associated mechanisms is the lowering of pain thresholds [47].

Sleep disorders and pain susceptibility can aggravate each other, suggesting a bidirectional and reciprocal relationship [48]; however, recent studies have pointed toward a stronger and more consistent unidirectional effect of sleep causing pain exacerbation [49,50,51].

Interestingly, sleep can be also disrupted by inflammatory processes. During the up-regulation of pro-inflammatory cytokines (including IL-1 and TNF), NREM sleep is increased [52,53], but also fragmented [54], while REM sleep is suppressed [55]. As a consequence, the lack of quality REM and non-REM sleep worsens body regeneration and increases pain sensitivity, which is believed to occur through dysregulation of the hypothalamus–pituitary–adrenal (HPA) axis and nociceptive perception [56]. Sleep is also altered during clinical conditions, such as depression [57]. Interestingly, pro-inflammatory cytokines and their mediators have also been shown to be associated with depression [58,59,60].

## 4. Inflammation–Psycho–Pain Axis

It is worth mentioning that not all cytokine depressive responses are equal, as different inflammatory profiles are associated with different sub-types of depression [61,62,63]. However, in general, it is known that pro-inflammatory cytokines are increased in major depressive disorder (MDD) [64,65,66,67], with the most remarkable increases in IL-6, TNF, and C-reactive protein (CRP) [68,69,70]. It has also been recently reported that IL-17 produced by gamma delta T-cells (gdTcells) may be a key link between the immune system and its effects on the body and mind, with particular emphasis on anxiety [71]. 

It is currently well-documented that depression is a strong predictor of orofacial pain and increased risk for the development of TMD [72,73,74,75,76]. This comorbidity may be due to overlapping pathways. Regions in the brain that are responsible for emotions send projections to pain modulation structures in the brainstem, which could explain why depression (a negative emotion) is often accompanied with an intensified pain response [77]. 

In 1977, Engel proposed the “biopsychosocial model” to address chronic pain in medicine [78]. This novel point of view led to the creation of the DC/TMD classification, including both a physical (Axis I) and a psychosocial (Axis II) appraisal [79]. Interestingly, the prognosis for patients with TMD is influenced more by Axis II factors than Axis I factors [80,81], emphasizing the importance of the psychological aspect in the pathogenesis of TMD. Knowing this, it would be reasonable to ask what the biological reasons for depression, anxiety, and increased pro-inflammatory cytokines are, in general. It turns out that our intestines—considered by some to be our “second brain” [82]—are becoming the prime suspect.

Tryptophan derived from dietary proteins can be metabolized into different substances, such as indole derivatives, which can induce anxiety. Tryptophan can also metabolize through the kynurenic pathway which, in microglia, is used to produce neurotoxic quinolinic acid (Quin), whereas astrocytes generate kynurenic acid (Kyna), which reduces neuronal excitability [83]. The third pathway of tryptophan metabolism leads to the production of serotonin, which has many positive effects in the CNS, but may also be a pain-inducing mediator that transmits pain signals directly to the vagus nerve [84]. Melatonin can be produced both in the gut, where it improves the function of microbiota, or in the pineal gland, where it regulates the circadian rhythm. Within the gut, bacteria such as *Lactobacillus* spp., *Bifidobacterium dentium*, and *Bifidobacterium* spp. [85] can also produce the inhibitory neurotransmitter GABA, which causes desensitization [86,87]. (Adapted from “Gut–Brain Axis”, by BioRender.com Retrieved from https://app.biorender.com/biorender-templates) (accessed on 1 January 2022).

## 5. Gut–Psycho–Pain Axis

**Figure 1:**
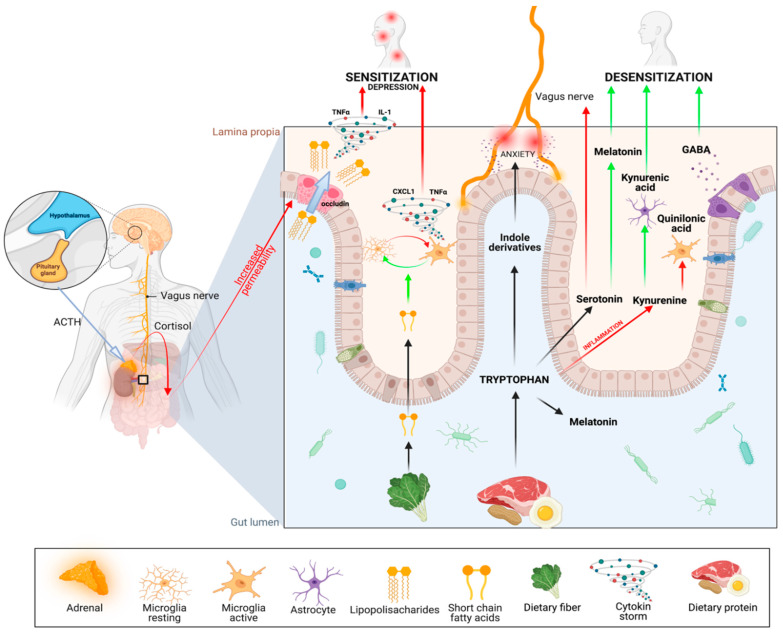
Communication between the gut microbiota and the central nervous system (CNS) in orofacial pain.Within the nervous system, stress can activate the HPA (hypothalamus–pituitary–adrenal) axis response, triggering the release of adrenocorticotrophic hormone (ACTH), which then initiates the synthesis and release of cortisol. Cortisol, in turn, affects intestinal barrier integrity by decreasing occludin levels. Several types of cells in the brain (e.g., microglia, astrocytes) are able to receive signals from the periphery, including the gastrointestinal tract [92,93,94]. Activation of these cells by lipopolysaccharides contributes to the development of depression and neuritis, which is one of the major mechanisms underlying the central sensitization associated with chronic pain [95,96]. Short-chain fatty acids derived from dietary fiber play an important regulatory role in activating microglia, thus protecting from sensitization.

The emerging role of the gut microbiota in neurological and psychiatric disorders has recently been demonstrated [88]. The whole cascade often starts with emotional stress, which can increase intestinal permeability, allowing bacteria to move across the intestinal mucosa [89] and causing visceral hyperalgesia (Figure 1). This happens as corticosteroids increase intestinal permeability through decreased levels of claudin-1, occludin, and zona occludens-1 [21]. Knowing that TMD has been associated with gastrointestinal disorders, especially IBS [90,91], it seems reasonable to ask whether the gut microbiota—in addition to its influence on the psyche—also has a direct influence on the perception of pain.

**Figure 2:**
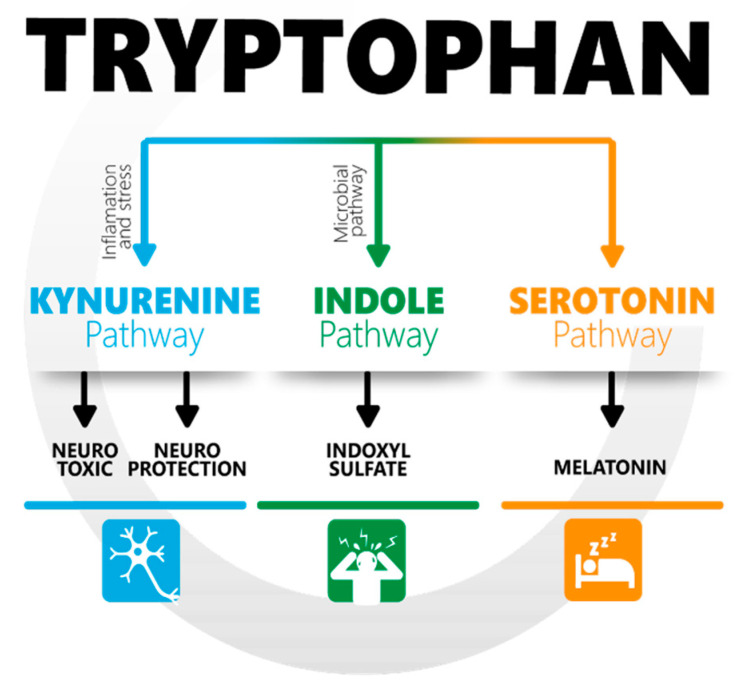
Tryptophan is processed by three different metabolic pathways, depending on the presence or absence of inflammatory process, stress, and actual requirements of the host (own resources).

It turns out that increased gut permeability, caused by cortisol, opens the gate for bacterial cell-wall components (e.g., lipopolysaccharides; LPS) that bind to pattern recognition receptors (PRR) expressed on immune cells and sensory neurons located in dorsal root ganglia [97,98]. This mechanism is considered to be an important contributor to peripheral sensitization [99]. Another important cause of sensitization comes from dietary tryptophan (Trp) and its metabolites. Interestingly, the microbiota play a key role in this metabolism [100] (Figure 2), which has three main pathways:

1. Production of serotonin (5-HT). Surprisingly, 90% of serotonin is produced by Enterococcus spp. in the gut. Decreased serotonin levels are associated with depression and, as recently hypothesized, with severe sleep bruxism [101]. It is also directly connected with a lack of its derivative—melatonin—as discussed in the following section. It turns out that inflammatory processes in the guts, as well as stress, cause the depletion of serotonin by changing the metabolic pathway of Trp [102] to the second one—the kynurenine pathway (KP) [103].
2. Kynurenine may be both good and bad for pain perception, as it may be further metabolized through two different pathways: either to kynurenic acid (KYNA), which reduces neuronal excitability [104,105], or quinolinic acid (Quin), which is neurotoxic [83].
3. The third metabolic pathway for Trp involves the production of indoxyl sulfate, which is associated with anxiety. Strikingly, the gut microbiota is exclusively responsible for the conversion of tryptophan to indole derivatives [106]. This provides further evidence that gut microbiome disruption can directly contribute to neuropsychiatric disorders [107], and that the link between our emotional state and microbiota is bidirectional [108,109]: stress disrupts the gut microbiome which, in turn, can cause stress.

Growing evidence has also suggested that disturbance of the gut microbiota significantly influences microglia maturation [110,111,112], the main functions of which are self-renewal to maintain CNS homeostasis and rapid responses to damage or infection [113]. However, prolonged activation of microglia contributes to disruption of the synthesis, reuptake, and release of neurotransmitters [114,115,116], as well as excessive production of pro-inflammatory cytokines, and can result in increased synaptic glutaminergic neurotransmission and decreased GABAergic synaptic neurotransmission [117,118,119,120]. This, in turn, contributes to the development of central sensitization and affects mood and cognition [121], with a particular emphasis on depression [122,123,124].

GERD affects the gastro-intestinal tract, but seems to also play a large role in terms of gut dysbiosis and pain. As GERD is associated with teeth grinding or clenching [125,126,127,128,129] and an almost three times higher risk of TMD, it is important to understand whether it is the disease itself or the side-effects of proton pump inhibitor (PPI) treatment that contributes to orofacial pain. Indeed, recent evidence has shown that pharmacological treatment of acid reflux could be more harmful than the disease itself. Some drugs for GERD treatment can negatively influence many intestinal processes [130], impair magnesium absorption [131,132,133], and contribute to malabsorption of vitamin B12 [134,135,136], which is manifested by neuropathy and mood disorders including personality change, psychosis, and emotional lability [137]. In addition, as stomach acid levels decline, more bacterial types can survive in the stomach and enter the small intestine, which can cause significant changes in the gut microbiota composition [138,139,140,141,142]. Moreover, by disrupting the bacterial flora, these drugs have been associated with problems such as irritable bowel syndrome [143,144], small intestinal bacterial overgrowth (SIBO) [145,146,147], and even stomach cancer [148,149,150,151]. The study of such possible negative consequences of PPIs has led to a search for alternative methods to treat GERD, such as melatonin supplementation [152,153,154,155].

Thus, knowing the possible metabolic mechanisms leading to sensitization, we should search for therapeutics that are anti-inflammatory, improve the function of the gut microbiome and, consequently, improve sleep and mood. There are at least five products that seems to fulfill these expectations, in terms of orofacial pain:

4. Probiotics (e.g., *Bacteroides fragilis*) can correct some of the changes related to increased gut permeability [156]. Probiotic supplementation has also shown promising results, with reductions in anxiety and depression [157,158,159,160] through direct and indirect mechanisms of action; for example, local stimulation of the vagus nerve [161,162,163,164,165], which reduces the activity of the sympathetic nervous system. Considering the potential beneficial effects of probiotics on mental health, they are also referred to as “psychobiotics” [83].
5. Omega-3 fatty acids show anti-inflammatory effects on LPS-stimulated microglia [166], induce an increase in several short-chain fatty acid-producing bacteria species [167], and help to maintain intestinal wall integrity. Thanks to those properties, they have been shown to be effective in the treatment of gut dysbiosis [168] (disruption to the microbiota homeostasis), depression [169], neuropathic pain [170] after neurotrauma [171], joint pain associated with rheumatoid arthritis, and inflammatory bowel disease [172].
6. Resveratrol is antioxidative, anti-inflammatory [173], and improves the gut microbiota [174]. Recently, resveratrol has been used to alleviate temporomandibular joint inflammatory pain by recovering disturbed gut microbiota. An interesting observation is that the systemic administration of resveratrol restored reduced Bacteroidetes and Lachnospiraceae while attenuating nociception in TMJ-inflamed mice, where the antinociceptive effect was mimicked by fecal transplantation from inflamed animals receiving resveratrol treatment [175].
7. Short-chain fatty acids (SCFAs), which are derived from bacterial fermentation of dietary fiber in the gut [176], play important roles in regulating microglia morphology and function. SCFAs may act as important mediators derived from the gut microbiota for regulation of pain through receptor-mediated mechanisms, epigenetic regulation mechanisms, or both [177,178,179,180].
8. The last therapeutic that should be discussed in this review is melatonin, which deserves a separate section.

## 6. Role of Melatonin in Pain, Sleep, and Inflammation

**Figure 3:**
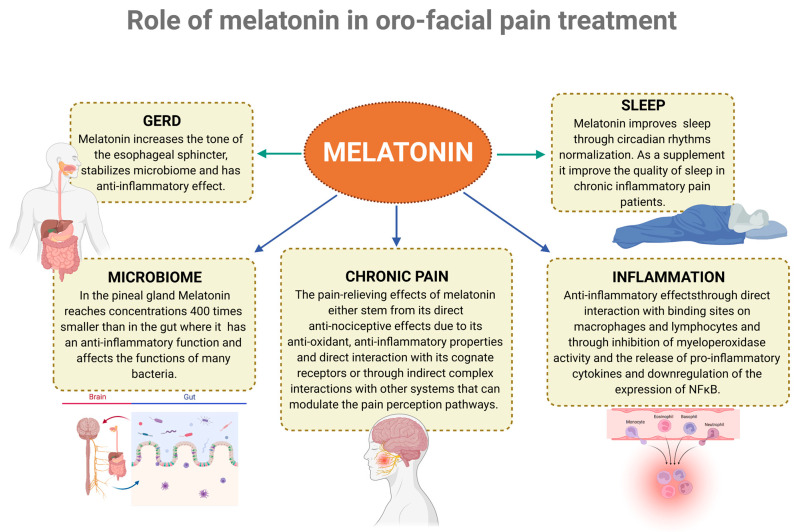
Melatonin improves the function of many processes that modulate or predict the presence of orofacial pain (Created with BioRender.com). GERD [189], Microbiome [190,191], Chronic pain [192,193], inflammation [194,195], sleep [192].

Melatonin, acting as a pleiotropic hormone, is released from the pineal gland and extra-pineal tissues, and plays a critical role in regulating the circadian rhythms [181]. Melatonin is largely produced in the intestines, where it reaches concentrations 400 times greater than that in the pineal gland and up to 100 times greater than that in the blood [182,183]. Initially, it was described as a sleep hormone, as it is secreted in the dark and induces sleep; however, it is now widely appreciated that it presents a wide array of activities (see Figure 3), encompassing anti-oxidant, anti-inflammatory, anti-apoptotic, anti-sympathetic nerve activation, endothelial cell preservation, neuroprotection, hepatoprotection, immunomodulation, thermoregulation, mood, and sexual behavior modulation [184,185,186,187,188]. 

Melatonin’s effect on pain is consistent with previous clinical and experimental data [196,197,198]. Regarding a low intensity of pain perception during the night, the possible analgesic effect of high melatonin during the night has been proposed as an associated mechanism [199]. Zhu et al., in a systematic review and meta-analysis of 19 randomized controlled trials using melatonin for various types of pain, reported a significant reduction in pain [200]. A recent meta-analysis of randomized, double-blind, and placebo-controlled trials concluded that melatonin might be used for the treatment of chronic pain, specifically endometriosis, IBS, and migraines [201].

Thus, melatonin may decrease pain by improving sleep through circadian rhythm normalization [202], but also through its own action on melatonin receptors and several neurotransmitter systems [193]. Animal models have also demonstrated that the suppression of melatonin secretion due to sleep deprivation can increase glial activation and aggravate neuropathic pain [203]. Moreover, disrupted melatonin secretion has been related to clinical symptoms in major depression and fibromyalgia patients [204].

Even national organizations on sleep research have started to recommend melatonin for insomnia symptoms, as well as in mood disorders, fibromyalgia, irritable bowel syndrome, functional dyspeptic syndrome, and temporomandibular joint dysfunction [192].

A recent double-blind, randomized, placebo-controlled study demonstrated that melatonin produces a reduction in overall pain, compared with placebo, in the treatment of myofascial TMD pain. In addition, it seems that the effect of melatonin on pain may be independent of the improvement in sleep quality. This conclusion is clinically relevant, as it suggests that its use does not need to be restricted to patients with pain and sleep disturbances [205].

There has been only one single case study relating bruxism with melatonin. A 7-year-old girl with sleep bruxism and sleep talking was managed with melatonin at 1.5 mg/day, with good results in two weeks and no adverse effects [206].

Additionally, a recent systematic review and meta-analysis of clinical trials indicated that interventions longer than 12 weeks and at a dosage of ≥10 mg/day were more efficacious in attenuating IL-6 and TNF-α levels, showing that long-term interventions with high doses of melatonin are required to effectively reduce inflammation [207]. From the available studies, doses of 10 mg (increasing blood levels up to 60-fold) [208] appear to be safe [209,210] and non-addictive [211].

The influence of melatonin on sex hormones has also become clearer than in the past. In men, clinical trials have indicated no differences in the hormonal synthesis of luteotropic hormone (LH), follicle-stimulating hormone (FSH), and testosterone after melatonin administration [212,213]. To the contrary, in women, treatment with melatonin decreased luteotropic hormone, estradiol, and progesterone [214,215,216]. Moreover, its potential to act directly on the epithelial mammary cells designates melatonin as a selective estrogen enzyme modulator [217,218]. The ability of melatonin to modulate estrogen appears to be particularly important in considering the sexual dimorphism of pain perception.

## 7. A proposed Cascade of Events Leading to Orofacial Pain and Gender Predisposition

There are multiple vulnerability factors that may perpetuate pain and facilitate the transition to chronic conditions, with a certain degree of unpredictability at the individual level [219].

An example case of a female patient with a history of PTSD can be used to depict a possible cascade of events. Importantly, the described cascade is by no means limited to people with PTSD which, in this case, is simply used for the sake of discussing the possible cascade of events and sex differences for risk factors.

PTSD is associated with a 2.56-fold higher risk of TMJ pain and 3.86-fold higher risk of muscle pain, with both types of pain being significantly higher for women [220]. Moreover, the risk of PTSD across the lifetime is also significantly higher for women than for men [221,222]. Interestingly, major depression is nearly two times more likely to occur in women than men [223], and women also show a higher inflammatory response to acute stress than men [224]. Based on the results from an 11-year follow-up study on Finnish adults, the effect of depressive symptoms on temporomandibular pain was also more direct in women [77]. Overall depression, anxiety, and somatoform disorders are all more prevalent in women than in men; however, the specific biological mechanisms contributing to such sex differences have only recently been discovered. The serotonin transporter (SERT), encoded by the SLC6A4 gene, turned out to be one of the causative mechanisms by which women exhibit an increased prevalence of somatic symptoms [225].

During PTSD, due to chronic stress, the gut epithelial layer become more permeable, leading to an increased movement of endotoxins and resulting in a low-grade inflammation and progressive tendency to lowered psychological mood. It is likely that, for this reason, trauma in childhood is associated with elevated levels of pro-inflammatory cytokines in later life [226]. Stress and inflammatory processes change the metabolic pathway of tryptophan towards the kyneurenic pathway. This, in turn, decreases levels of serotonin, with all of the possible consequences associated with depression, sleep bruxism [101], and dysregulation of the circadian rhythm due to a lack of melatonin. 

Women are also 41% more likely than men to experience insomnia [227]. Moreover, acute sleep loss leads to alterations in inflammatory gene expression [228], which, in women, showed greater up-regulation compared to that in men [229]. Inflammatory cytokines may directly disrupt sleep, but may also be associated with depression, which changes the sleep composition even further. As a consequence, patients lacking REM sleep are likely to be more emotionally reactive. Their NON-REM sleep is fragmented, causing higher anxiety, fatigue, and increased pain sensitivity.

In short, the worse the sleep, the more the pain and stress. Conversely, in the case of our hypothetical patient with PTSD, the higher the stress level, the more extreme the gut dysbiosis and the worse the sleep quality.

In these conditions, it is quite easy to get used to substances that give relief, as a chronic lack of sleep is conducive to addiction [230,231,232,233]; in particular, PTSD significantly increases the risk of alcohol use disorder [234]. Generally, individuals who are particularly sensitive to stress drink alcohol to fall asleep [235]; however, this habit makes sleeping difficult and worsens sleep apnea [236], especially if it was already present. Indeed, alcohol before bedtime causes sleep fragmentation and, notably, additionally reduces REM sleep [237]. Notwithstanding, if the patient is diagnosed with depression and treated with antidepressants, such as SSRIs, these drugs may carry the risk of depriving someone of REM sleep and its beneficial effects on the regulation of emotions [238]. Interestingly, daily sleepiness after poor quality sleep stimulates over-eating with unhealthy food [239], which may affect the tone of the esophageal sphincter leading to GERD. Reflux symptoms affect women more than men [240]. Additionally, physiological stress increases the perception of heartburn and aggravates GERD symptoms, increasing the need for treatment [241].

**Figure 4:**
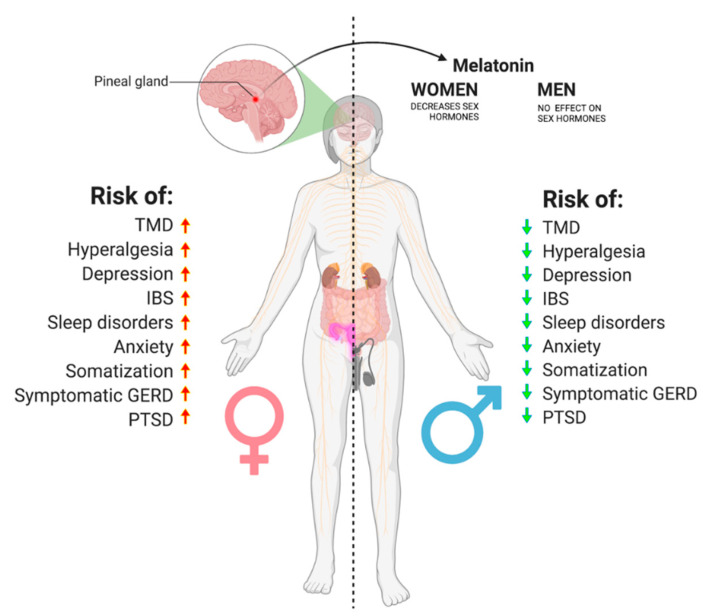
All of the TMD risk factors discussed in this review appear to be much more common in women. Moreover, melatonin seems to affect female sex hormones, while not influencing male testosterone (Created with BioRender.com) (accessed on 1 January 2022).

During GERD treatment, PPI-driven gastric hypochlorhydria can modify the composition of the gut microbiota, adding to the stress-mediated changes. As described in the preceding sections, dysbiosis can directly modulate the neuronal excitability, contributing to many types of chronic pain. This may partially explain the findings that both GERD and IBS are associated with a 3 times higher risk of TMD. However, this mechanistic explanation should be considered as part of a bigger picture, knowing the relation between GERD and psychological disorders [242,243,244] and that between psychological factors and the onset and persistence of TMD [245]. Sex hormones also seem to play an important role in this case. Estradiol directly increases the number of Proteobacteria species and decreases Prevotellaceae in females, causing increased LPS (lipopolysaccharide) and decreased SCFA production, respectively, thereby increasing the risk of mental disorders in the pubertal and reproductive phases [246,247,248]. Moreover, oral contraceptives and ovariectomy are also associated with changes in the gut microbiota [249]. All of these mentioned risk factors are higher for women (Figure 4), likely mediated by genetic predispositions and the fluctuation of sex hormones. 

Therefore, it should not be surprising that the occurrence of malocclusion, occlusal interferences, and missing teeth is nearly equal for males and females [250], while it is known that the risk of TMD in women is more than twice as high [251], as well as the need for treatment. 

**Figure 5:**
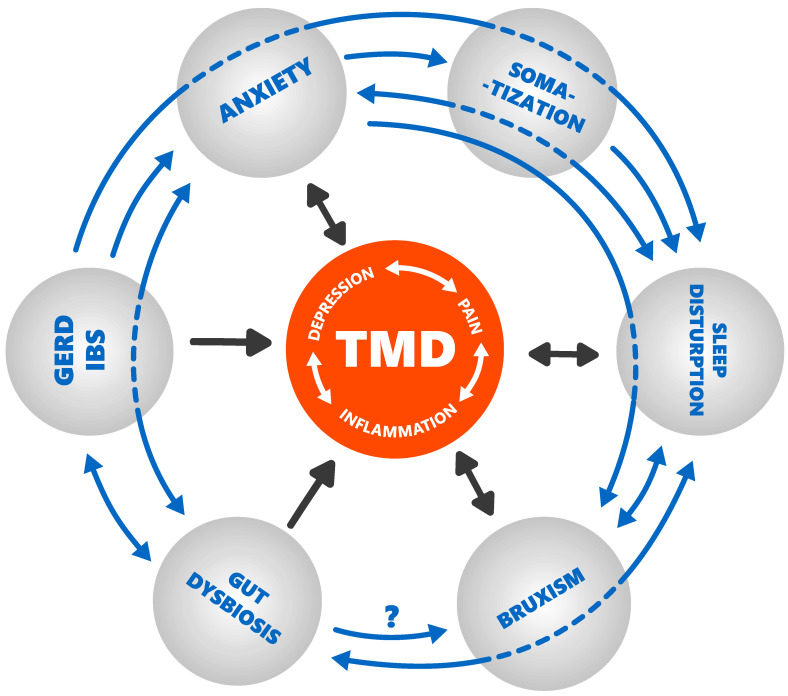
Vicious cycle of chronic diseases leading to chronic temporomandibular disorders (own resources).

As a final result, patients may take several medications for IBS, GERD, depression, anxiety, insomnia, pain, neuropathy, inflammation, and muscle soreness, contributing to the perpetuation of an interactive viscous circle of chronic diseases (Figure 5). This may be a proposed mechanism to link the various conditions/phenomena. In this vicious cycle, it is not possible to state with certainty where it begins yet; at any rate, it is likely that the primary condition differs at the individual level. Based on this, future research on the interactions of factors implicated in the development of the gut–brain axis and their influence on pain, mood, and sleep modulation is recommended.

## 8. Conclusions

From this review emerged the fact that inflammation, through disruption of the sleep cycle, may worsen body regeneration and increase pain sensitivity, anxiety, and stress. This, in turn, increases intestinal permeability and disrupts the microbiota, leading (directly and indirectly) to sensitization of the central nervous system, nutrition malabsorption, and hyperactivation of microglia, possibly contributing to many types of chronic pain, including visceral, inflammatory, headache, and neuropathic pain. The gut microbiome can be also negatively altered by GERD, especially during treatment with PPIs. Inflammation and disruption of the intestinal microbiome alter the metabolism of tryptophan and its important derivatives, serotonin and melatonin, both of which seem to be crucial regulators of pathophysiology in the treatment of chronic orofacial pain. GERD, IBS, sleep disorders, anxiety, depression, PTSD, hyperalgesia, and somatization are all more prevalent among women than men, and so are TMDs. This might further support the hypothesis of the existence of a Gut–Sleep–Psycho–TMD axis.

Key Findings

- Inflammation negatively influences the sleep cycle, leading to a pre-disposition to higher pain sensitivity, anxiety, and stress and, as consequence, to intestinal permeability and disrupted microbiota;
- Bacterial dysbiosis leads (directly and indirectly) to sensitization of the central nervous system, possibly contributing to many types of chronic pain;
- Inflammation and disruption of the intestinal microbiome alter the metabolism of serotonin and melatonin;
- GERD, IBS, sleep disorders, anxiety, depression, PTSD, hyperalgesia, and somatization are all more prevalent among women than men, and so are TMDs.

## Data Availability

Not applicable.

## Abbreviations

TMD	Temporomandibular disorder
TMJ	Temporomandibular joint
CNS	Central Nervous System
GERD	Gastroesophageal reflux disease
IBS	Irritable Bowel Syndrome
SIBO	Small intestinal bacterial overgrowth
PTSD	Post-traumatic stress disorder
REM	Rapid eye movement
Non-REM SWS	Non-rapid eye movement slow-wave sleep
PPI	Proton pump inhibitor
SCFA	Short-Chain Fatty Acids
LPS	Lipopolysaccharides
GABA	Gamma-aminobutyric acid
LH	Luteinizing hormone
FSH	Follicle-stimulating hormone
HPA	Hypothalamic–pituitary–adrenal
MDD	Major depressive disorder
gdTcells	Gamma delta T-cells
CRP	C-reactive protein
ACTH	Adrenocorticotropic hormone
KP	Kynurenine pathway
Kyna	Kynurenic acid
Quin	Quinolinic acid
PRR	Pattern recognition receptors
Trp	Tryptophan
5-HT	serotonin

## References

[1] Scrivani S.J., Keith D.A., Kaban L.B. (2008). Temporomandibular Disorders. N. Engl. J. Med..

[2] Macfarlane T.V., Glenny A.-M., Worthington H.V. (2001). Systematic Review of Population-Based Epidemiological Studies of Oro-Facial Pain. J. Dent..

[3] Truelove E.L., Sommers E.E., LeResche L., Dworkin S.F., von Korff M. (1992). Clinical Diagnostic Criteria for TMD New Classification Permits Multiple Diagnoses. J. Am. Dent. Assoc..

[4] Dworkin S.F., LeResche L. (1992). Research Diagnostic Criteria for Temporomandibular Disorders: Review, Criteria, Examinations and Specifications, Critique. J. Craniomandib. Disord..

[5] Munzenmaier D.H., Wilentz J., Cowley A.W. (2014). Genetic, Epigenetic, and Mechanistic Studies of Temporomandibular Disorders and Overlapping Pain Conditions. Mol. Pain.

[6] Gatchel R.J., Reuben D.B., Dagenais S., Turk D.C., Chou R., Hershey A.D., Hicks G.E., Licciardone J.C., Horn S.D. (2018). Research Agenda for the Prevention of Pain and Its Impact: Report of the Work Group on the Prevention of Acute and Chronic Pain of the Federal Pain Research Strategy. J. Pain.

[7] Gallotta S., Bruno V., Catapano S., Mobilio N., Ciacci C., Iovino P. (2017). High Risk of Temporomandibular Disorder in Irritable Bowel Syndrome: Is There a Correlation with Greater Illness Severity?. World J. Gastroenterol..

[8] Versteeg R.I., Serlie M.J., Kalsbeek A., la Fleur S.E. (2015). Serotonin, a Possible Intermediate between Disturbed Circadian Rhythms and Metabolic Disease. Neuroscience.

[9] Clarke G., Grenham S., Scully P., Fitzgerald P., Moloney R.D., Shanahan F., Dinan T.G., Cryan J.F. (2013). The Microbiome-Gut-Brain Axis during Early Life Regulates the Hippocampal Serotonergic System in a Sex-Dependent Manner. Mol. Psychiatry.

[10] Ridaura V., Belkaid Y. (2015). Gut Microbiota: The Link to Your Second Brain. Cell.

[11] Guo R., Chen L.-H., Xing C., Liu T. (2019). Pain Regulation by Gut Microbiota: Molecular Mechanisms and Therapeutic Potential. Br. J. Anaesth..

[12] Staffe A.T., Bech M.W., Clemmensen S.L.K., Nielsen H.T., Larsen D.B., Petersen K.K. (2019). Total Sleep Deprivation Increases Pain Sensitivity, Impairs Conditioned Pain Modulation and Facilitates Temporal Summation of Pain in Healthy Participants. PLoS ONE.

[13] Herrero Babiloni A., de Koninck B.P., Beetz G., de Beaumont L., Martel M.O., Lavigne G.J. (2020). Sleep and Pain: Recent Insights, Mechanisms, and Future Directions in the Investigation of This Relationship. J. Neural Transm..

[14] Stroemel-Scheder C., Kundermann B., Lautenbacher S. (2020). The Effects of Recovery Sleep on Pain Perception: A Systematic Review. Neurosci. Biobehav. Rev..

[15] Al-Jewair T., Shibeika D., Ohrbach R. (2021). Temporomandibular Disorders and Their Association with Sleep Disorders in Adults: A Systematic Review. J. Oral Facial Pain Headache.

[16] Rener-Sitar K., John M.T., Pusalavidyasagar S.S., Bandyopadhyay D., Schiffman E.L. (2016). Sleep Quality in Temporomandibular Disorder Cases. Sleep Med..

[17] Sommer I., Lavigne G., Ettlin D.A. (2015). Review of Self-Reported Instruments That Measure Sleep Dysfunction in Patients Suffering from Temporomandibular Disorders and/or Orofacial Pain. Sleep Med..

[18] Smith M.T., Wickwire E.M., Grace E.G., Edwards R.R., Buenaver L.F., Peterson S., Klick B., Haythornthwaite J.A. (2009). Sleep Disorders and Their Association with Laboratory Pain Sensitivity in Temporomandibular Joint Disorder. Sleep.

[19] Benoliel R., Zini A., Zakuto A., Slutzky H., Haviv Y., Sharav Y., Almoznino G. (2017). Subjective Sleep Quality in Temporomandibular Disorder Patients and Association with Disease Characteristics and Oral Health–Related Quality of Life. J. Oral Facial Pain Headache.

[20] van der Helm E., Yao J., Dutt S., Rao V., Saletin J.M., Walker M.P. (2011). REM Sleep Depotentiates Amygdala Activity to Previous Emotional Experiences. Curr. Biol..

[21] Zheng G., Wu S.-P., Hu Y., Smith D.E., Wiley J.W., Hong S. (2013). Corticosterone Mediates Stress-Related Increased Intestinal Permeability in a Region-Specific Manner. Neurogastroenterol. Motil..

[22] Cussotto S., Sandhu K.V., Dinan T.G., Cryan J.F. (2018). The Neuroendocrinology of the Microbiota-Gut-Brain Axis: A Behavioural Perspective. Front. Neuroendocrinol..

[23] Neckelmann D., Mykletun A., Dahl A.A. (2007). Chronic Insomnia as a Risk Factor for Developing Anxiety and Depression. Sleep.

[24] Breslau N., Roth T., Rosenthal L., Andreski P. (1996). Sleep Disturbance and Psychiatric Disorders: A Longitudinal Epidemiological Study of Young Adults. Biol. Psychiatry.

[25] Papadimitriou G.N., Linkowski P. (2005). Sleep Disturbance in Anxiety Disorders. Int. Rev. Psychiatry.

[26] Kessler R.C., Aguilar-Gaxiola S., Alonso J., Chatterji S., Lee S., Ormel J., Üstün T.B., Wang P.S. (2009). The Global Burden of Mental Disorders: An Update from the WHO World Mental Health (WMH) Surveys. Epidemiol. Psichiatr. Soc..

[27] Dang-Vu T.T., Schabus M., Desseilles M., Sterpenich V., Bonjean M., Maquet P. (2010). Functional Neuroimaging Insights into the Physiology of Human Sleep. Sleep.

[28] Miyauchi S., Misaki M., Kan S., Fukunaga T., Koike T. (2009). Human Brain Activity Time-Locked to Rapid Eye Movements during REM Sleep. Exp. Brain Res..

[29] Nofzinger E.A. (2005). Functional Neuroimaging of Sleep. Semin. Neurol..

[30] McGinty D.J., Harper R.M. (1976). Dorsal Raphe Neurons: Depression of Firing during Sleep in Cats. Brain Res..

[31] Marrosu F., Portas C., Mascia M.S., Casu M.A., Fà M., Giagheddu M., Imperato A., Gessa G.L. (1995). Microdialysis Measurement of Cortical and Hippocampal Acetylcholine Release during Sleep-Wake Cycle in Freely Moving Cats. Brain Res..

[32] Kametani H., Kawamura H. (1990). Alterations in Acetylcholine Release in the Rat Hippocampus during Sleep-Wakefulness Detected by Intracerebral Dialysis. Life Sci..

[33] Shouse M.N., Staba R.J., Saquib S.F., Farber P.R. (2000). Monoamines and Sleep: Microdialysis Findings in Pons and Amygdala. Brain Res..

[34] Park S.P. (2002). In Vivo Microdialysis Measures of Extracellular Norepinephrine in the Rat Amygdala during Sleep-Wakefulness. J. Korean Med. Sci..

[35] Ouyang M., Hellman K., Abel T., Thomas S.A. (2004). Adrenergic Signaling Plays a Critical Role in the Maintenance of Waking and in the Regulation of REM Sleep. J. Neurophysiol..

[36] Walker M.P., van der Helm E. (2009). Overnight Therapy? The Role of Sleep in Emotional Brain Processing. Psychol. Bull..

[37] Levin R., Nielsen T.A. (2007). Disturbed Dreaming, Posttraumatic Stress Disorder, and Affect Distress: A Review and Neurocognitive Model. Psychol. Bull..

[38] ben Simon E., Rossi A., Harvey A.G., Walker M.P. (2020). Overanxious and Underslept. Nat. Hum. Behav.

[39] Goldstein A.N., Walker M.P. (2014). The Role of Sleep in Emotional Brain Function. Annu. Rev. Clin. Psychol..

[40] Ekman P., Davidson R.J. (1994). The Nature of Emotion: Fundamental Questions.

[41] Lautenbacher S., Kundermann B., Krieg J. (2006). Sleep Deprivation and Pain Perception. Sleep Med. Rev..

[42] Schuh-Hofer S., Wodarski R., Pfau D.B., Caspani O., Magerl W., Kennedy J.D., Treede R.-D. (2013). One Night of Total Sleep Deprivation Promotes a State of Generalized Hyperalgesia: A Surrogate Pain Model to Study the Relationship of Insomnia and Pain. Pain.

[43] Faraut B., Léger D., Medkour T., Dubois A., Bayon V., Chennaoui M., Perrot S. (2015). Napping Reverses Increased Pain Sensitivity Due to Sleep Restriction. PLoS ONE.

[44] Roehrs T., Hyde M., Blaisdell B., Greenwald M., Roth T. (2006). Sleep Loss and REM Sleep Loss Are Hyperalgesic. Sleep.

[45] Lentz M.J., Landis C.A., Rothermel J., Shaver J.L. (1999). Effects of Selective Slow Wave Sleep Disruption on Musculoskeletal Pain and Fatigue in Middle Aged Women. J. Rheumatol..

[46] Azevedo E., Manzano G.M., Silva A., Martins R., Andersen M.L., Tufik S. (2011). The Effects of Total and REM Sleep Deprivation on Laser-Evoked Potential Threshold and Pain Perception. Pain.

[47] Schrimpf M., Liegl G., Boeckle M., Leitner A., Geisler P., Pieh C. (2015). The Effect of Sleep Deprivation on Pain Perception in Healthy Subjects: A Meta-Analysis. Sleep Med..

[48] Cheatle M.D., Foster S., Pinkett A., Lesneski M., Qu D., Dhingra L. (2016). Assessing and Managing Sleep Disturbance in Patients with Chronic Pain. Anesthesiol. Clin..

[49] Finan P.H., Goodin B.R., Smith M.T. (2013). The Association of Sleep and Pain: An Update and a Path Forward. J. Pain.

[50] Lavigne G.J., Sessle B.J. (2016). The Neurobiology of Orofacial Pain and Sleep and Their Interactions. J. Dent. Res..

[51] Andersen M.L., Araujo P., Frange C., Tufik S. (2018). Sleep Disturbance and Pain. Chest.

[52] Krueger J.M., Obál F., Fang J., Kubota T., Taishi P. (2006). The Role of Cytokines in Physiological Sleep Regulation. Ann. N. Y. Acad. Sci..

[53] Opp M.R. (2005). Cytokines and Sleep. Sleep Med. Rev..

[54] Olivadoti M.D., Opp M.R. (2008). Effects of i.c.v. Administration of Interleukin-1 on Sleep and Body Temperature of Interleukin-6-Deficient Mice. Neuroscience.

[55] Krueger J.M., Majde J.A. (1994). Microbial Products and Cytokines in Sleep and Fever Regulation. Crit. Rev. Immunol..

[56] de Oliveira C.M.B., Sakata R.K., Issy A.M., Gerola L.R., Salomão R. (2011). Cytokines and Pain. Braz. J. Anesthesiol..

[57] Anisman H., Merali Z., Hayley S. (2008). Neurotransmitter, Peptide and Cytokine Processes in Relation to Depressive Disorder: Comorbidity between Depression and Neurodegenerative Disorders. Prog. Neurobiol..

[58] Tabatabaeizadeh S.-A., Abdizadeh M.F., Meshkat Z., Khodashenas E., Darroudi S., Fazeli M., Ferns G.A., Avan A., Ghayour-Mobarhan M. (2018). There Is an Association between Serum High-Sensitivity C-Reactive Protein (Hs-CRP) Concentrations and Depression Score in Adolescent Girls. Psychoneuroendocrinology.

[59] Zalli A., Jovanova O., Hoogendijk W.J.G., Tiemeier H., Carvalho L.A. (2016). Low-Grade Inflammation Predicts Persistence of Depressive Symptoms. Psychopharmacology.

[60] Parkinson J.T., Foley É.M., Jadon D.R., Khandaker G.M. (2020). Depression in Patients with Spondyloarthritis: Prevalence, Incidence, Risk Factors, Mechanisms and Management. Ther. Adv. Musculoskelet. Dis..

[61] Karlović D., Serretti A., Vrkić N., Martinac M., Marčinko D. (2012). Serum Concentrations of CRP, IL-6, TNF-α and Cortisol in Major Depressive Disorder with Melancholic or Atypical Features. Psychiatry Res..

[62] Kaestner F., Hettich M., Peters M., Sibrowski W., Hetzel G., Ponath G., Arolt V., Cassens U., Rothermundt M. (2005). Different Activation Patterns of Proinflammatory Cytokines in Melancholic and Non-Melancholic Major Depression Are Associated with HPA Axis Activity. J. Affect. Disord..

[63] Dunjic-Kostic B., Ivkovic M., Radonjic N.V., Petronijevic N.D., Pantovic M., Damjanovic A., Poznanovic S.T., Jovanovic A., Nikolic T., Jasovic-Gasic M. (2013). Melancholic and Atypical Major Depression--Connection between Cytokines, Psychopathology and Treatment. Prog. Neuropsychopharmacol. Biol. Psychiatry.

[64] Köhler C.A., Freitas T.H., Maes M., de Andrade N.Q., Liu C.S., Fernandes B.S., Stubbs B., Solmi M., Veronese N., Herrmann N. (2017). Peripheral Cytokine and Chemokine Alterations in Depression: A Meta-Analysis of 82 Studies. Acta Psychiatr. Scand..

[65] Liu Y., Ho R.C.-M., Mak A. (2012). Interleukin (IL)-6, Tumour Necrosis Factor Alpha (TNF-α) and Soluble Interleukin-2 Receptors (SIL-2R) Are Elevated in Patients with Major Depressive Disorder: A Meta-Analysis and Meta-Regression. J. Affect. Disord..

[66] Howren M.B., Lamkin D.M., Suls J. (2009). Associations of Depression with C-Reactive Protein, IL-1, and IL-6: A Meta-Analysis. Psychosom. Med..

[67] Dowlati Y., Herrmann N., Swardfager W., Liu H., Sham L., Reim E.K., Lanctôt K.L. (2010). A Meta-Analysis of Cytokines in Major Depression. Biol. Psychiatry.

[68] Stewart J.C., Rand K.L., Muldoon M.F., Kamarck T.W. (2009). A Prospective Evaluation of the Directionality of the Depression-Inflammation Relationship. Brain Behav. Immun..

[69] Miller A.H., Maletic V., Raison C.L. (2009). Inflammation and Its Discontents: The Role of Cytokines in the Pathophysiology of Major Depression. Biol. Psychiatry.

[70] Maes M., Yirmyia R., Noraberg J., Brene S., Hibbeln J., Perini G., Kubera M., Bob P., Lerer B., Maj M. (2009). The Inflammatory & Neurodegenerative (I&ND) Hypothesis of Depression: Leads for Future Research and New Drug Developments in Depression. Metab. Brain Dis..

[71] Alves de Lima K., Rustenhoven J., da Mesquita S., Wall M., Salvador A.F., Smirnov I., Martelossi Cebinelli G., Mamuladze T., Baker W., Papadopoulos Z. (2020). Meningeal Γδ T Cells Regulate Anxiety-like Behavior via IL-17a Signaling in Neurons. Nat. Immunol..

[72] Liao C.-H., Chang C.-S., Chang S.-N., Lane H.-Y., Lyu S.-Y., Morisky D.E., Sung F.-C. (2011). The Risk of Temporomandibular Disorder in Patients with Depression: A Population-Based Cohort Study. Community Dent. Oral Epidemiol..

[73] Slade G.D., Diatchenko L., Bhalang K., Sigurdsson A., Fillingim R.B., Belfer I., Max M.B., Goldman D., Maixner W. (2007). Influence of Psychological Factors on Risk of Temporomandibular Disorders. J. Dent. Res..

[74] Nevalainen N., Lähdesmäki R., Mäki P., Ek E., Taanila A., Pesonen P., Sipilä K. (2017). Association of Stress and Depression with Chronic Facial Pain: A Case-Control Study Based on the Northern Finland 1966 Birth Cohort. CRANIO®.

[75] Sipilä K., Mäki P., Laajala A., Taanila A., Joukamaa M., Veijola J. (2013). Association of Depressiveness with Chronic Facial Pain: A Longitudinal Study. Acta Odontol. Scand..

[76] Korszun A., Hinderstein B., Wong M., Peterson L.J. (1996). Comorbidity of Depression with Chronic Facial Pain and Temporomandibular Disorders. Oral Surg. Oral Med. Oral Pathol. Oral Radiol. Endodontol..

[77] Banafa A., Sipilä K., Suvisaari J., Suominen A.L. (2021). Low-Grade Inflammation as a Potential Mediator between Depressive Symptoms and Temporomandibular Pain: An 11-Year Follow-up Study on Finnish Adults. Acta Odontol. Scand..

[78] Engel G.L. (1977). The Need for a New Medical Model: A Challenge for Biomedicine. Science.

[79] Manfredini D., Ahlberg J., Winocur E., Guarda-Nardini L., Lobbezoo F. (2011). Correlation of RDC/TMD Axis I Diagnoses and Axis II Pain-Related Disability. A Multicenter Study. Clin. Oral Investig..

[80] Manfredini D., Favero L., del Giudice A., Masiero S., Stellini E., Guarda-Nardini L. (2013). Axis II Psychosocial Findings Predict Effectiveness of TMJ Hyaluronic Acid Injections. Int. J. Oral Maxillofac. Surg..

[81] Slade G.D., Ohrbach R., Greenspan J.D., Fillingim R.B., Bair E., Sanders A.E., Dubner R., Diatchenko L., Meloto C.B., Smith S. (2016). Painful Temporomandibular Disorder. J. Dent. Res..

[82] Ochoa-Repáraz J., Kasper L.H. (2016). The Second Brain: Is the Gut Microbiota a Link Between Obesity and Central Nervous System Disorders?. Curr. Obes. Rep..

[83] Müller N., Schwarz M.J. (2007). The Immune-Mediated Alteration of Serotonin and Glutamate: Towards an Integrated View of Depression. Mol. Psychiatry.

[84] Xu X., Chen R., Zhan G., Wang D., Tan X., Xu H. (2021). Enterochromaffin Cells: Sentinels to Gut Microbiota in Hyperalgesia?. Front. Cell. Infect. Microbiol..

[85] Sharon G., Sampson T.R., Geschwind D.H., Mazmanian S.K. (2016). The Central Nervous System and the Gut Microbiome. Cell.

[86] Du X., Hao H., Yang Y., Huang S., Wang C., Gigout S., Ramli R., Li X., Jaworska E., Edwards I. (2017). Local GABAergic Signaling within Sensory Ganglia Controls Peripheral Nociceptive Transmission. J. Clin. Investig..

[87] Pokusaeva K., Johnson C., Luk B., Uribe G., Fu Y., Oezguen N., Matsunami R.K., Lugo M., Major A., Mori-Akiyama Y. (2017). GABA-Producing *Bifidobacterium Dentium* Modulates Visceral Sensitivity in the Intestine. Neurogastroenterol. Motil..

[88] Bear T.L.K., Dalziel J.E., Coad J., Roy N.C., Butts C.A., Gopal P.K. (2020). The Role of the Gut Microbiota in Dietary Interventions for Depression and Anxiety. Adv. Nutr..

[89] Gareau M., Silva M., Perdue M. (2008). Pathophysiological Mechanisms of Stress-Induced Intestina Damage. Curr. Mol. Med..

[90] Whitehead W.E., Palsson O., Jones K.R. (2002). Systematic Review of the Comorbidity of Irritable Bowel Syndrome with Other Disorders: What Are the Causes and Implications?. Gastroenterology.

[91] Durham J., Newton-John T.R.O., Zakrzewska J.M. (2015). Temporomandibular Disorders. BMJ.

[92] Duan L., Zhang X.-D., Miao W.-Y., Sun Y.-J., Xiong G., Wu Q., Li G., Yang P., Yu H., Li H. (2018). PDGFRβ Cells Rapidly Relay In-flammatory Signal from the Circulatory System to Neurons via Chemokine CCL2. Neuron.

[93] Rafalski V.A., Merlini M., Akassoglou K. (2018). Pericytes: The Brain’s Very First Responders?. Neuron.

[94] Tang A.T., Choi J.P., Kotzin J.J., Yang Y., Hong C.C., Hobson N., Girard R., Zeineddine H.A., Lightle R., Moore T. (2017). Endothelial TLR4 and the Microbiome Drive Cerebral Cavernous Malformations. Nature.

[95] Tsuda M. (2018). Modulation of Pain and Itch by Spinal Glia. Neurosci. Bull..

[96] Ji R.-R., Xu Z.-Z., Gao Y.-J. (2014). Emerging Targets in Neuroinflammation-Driven Chronic Pain. Nat. Rev. Drug Discov..

[97] Das N., Dewan V., Grace P.M., Gunn R.J., Tamura R., Tzarum N., Watkins L.R., Wilson I.A., Yin H. (2016). HMGB1 Activates Proinflammatory Signaling via TLR5 Leading to Allodynia. Cell Rep..

[98] Miller R.E., Ishihara S., Tran P.B., Golub S.B., Last K., Miller R.J., Fosang A.J., Malfait A.-M. (2018). An Aggrecan Fragment Drives Osteoarthritis Pain through Toll-like Receptor 2. JCI Insight.

[99] Liu T., Gao Y.-J., Ji R.-R. (2012). Emerging Role of Toll-like Receptors in the Control of Pain and Itch. Neurosci. Bull..

[100] Ramos-Chávez L.A., Lugo Huitrón R., González Esquivel D., Pineda B., Ríos C., Silva-Adaya D., Sánchez-Chapul L., Roldán-Roldán G., Pérez de la Cruz V. (2018). Relevance of Alternative Routes of Kynurenic Acid Production in the Brain. Oxidative Med. Cell. Longev..

[101] Smardz J., Martynowicz H., Wojakowska A., Wezgowiec J., Danel D., Mazur G., Wieckiewicz M. (2022). Lower Serotonin Levels in Severe Sleep Bruxism and Its Association with Sleep, Heart Rate, and Body Mass Index. J. Oral Rehabil..

[102] Cervenka I., Agudelo L.Z., Ruas J.L. (2017). Kynurenines: Tryptophan’s Metabolites in Exercise, Inflammation, and Mental Health. Science.

[103] Stone T.W., Stoy N., Darlington L.G. (2013). An Expanding Range of Targets for Kynurenine Metabolites of Tryptophan. Trends Pharmacol. Sci..

[104] Resta F., Masi A., Sili M., Laurino A., Moroni F., Mannaioni G. (2016). Kynurenic Acid and Zaprinast Induce Analgesia by Modulating HCN Channels through GPR35 Activation. Neuropharmacology.

[105] Cosi C., Mannaioni G., Cozzi A., Carlà V., Sili M., Cavone L., Maratea D., Moroni F. (2011). G-Protein Coupled Receptor 35 (GPR35) Activation and Inflammatory Pain: Studies on the Antinociceptive Effects of Kynurenic Acid and Zaprinast. Neuropharmacology.

[106] Wikoff W.R., Anfora A.T., Liu J., Schultz P.G., Lesley S.A., Peters E.C., Siuzdak G. (2009). Metabolomics Analysis Reveals Large Effects of Gut Microflora on Mammalian Blood Metabolites. Proc. Natl. Acad. Sci. USA..

[107] Brydges C.R., Fiehn O., Mayberg H.S., Schreiber H., Dehkordi S.M., Bhattacharyya S., Cha J., Choi K.S., Craighead W.E., Krishnan R.R. (2021). Indoxyl Sulfate, a Gut Microbiome-Derived Uremic Toxin, Is Associated with Psychic Anxiety and Its Functional Magnetic Resonance Imaging-Based Neurologic Signature. Sci. Rep..

[108] Allen R.G., Lafuse W.P., Galley J.D., Ali M.M., Ahmer B.M.M., Bailey M.T. (2012). The Intestinal Microbiota Are Necessary for Stressor-Induced Enhancement of Splenic Macrophage Microbicidal Activity. Brain Behav. Immun..

[109] Cryan J.F., O’Riordan K.J., Cowan C.S.M., Sandhu K.v., Bastiaanssen T.F.S., Boehme M., Codagnone M.G., Cussotto S., Fulling C., Golubeva A.v. (2019). The Microbiota-Gut-Brain Axis. Physiol. Rev..

[110] Abdel-Haq R., Schlachetzki J.C.M., Glass C.K., Mazmanian S.K. (2019). Microbiome–Microglia Connections via the Gut–Brain Axis. J. Exp. Med..

[111] Erny D., Hrabě de Angelis A.L., Prinz M. (2017). Communicating Systems in the Body: How Microbiota and Microglia Cooperate. Immunology.

[112] Erny D., Hrabě de Angelis A.L., Jaitin D., Wieghofer P., Staszewski O., David E., Keren-Shaul H., Mahlakoiv T., Jakobshagen K., Buch T. (2015). Host Microbiota Constantly Control Maturation and Function of Microglia in the CNS. Nat. Neurosci..

[113] Yirmiya R., Rimmerman N., Reshef R. (2015). Depression as a Microglial Disease. Trends Neurosci..

[114] Stephan A.H., Barres B.A., Stevens B. (2012). The Complement System: An Unexpected Role in Synaptic Pruning during Development and Disease. Annu. Rev. Neurosci..

[115] Elmer B.M., McAllister A.K. (2012). Major Histocompatibility Complex Class I Proteins in Brain Development and Plasticity. Trends Neurosci..

[116] Deverman B.E., Patterson P.H. (2009). Cytokines and CNS Development. Neuron.

[117] Gao Y.-J., Ji R.-R. (2010). Chemokines, Neuronal–Glial Interactions, and Central Processing of Neuropathic Pain. Pharmacol. Ther..

[118] Gao Y.-J., Ji R.-R. (2010). Targeting Astrocyte Signaling for Chronic Pain. Neurotherapeutics.

[119] Chen G., Zhang Y.-Q., Qadri Y.J., Serhan C.N., Ji R.-R. (2018). Microglia in Pain: Detrimental and Protective Roles in Pathogenesis and Resolution of Pain. Neuron.

[120] Matsuda M., Huh Y., Ji R.-R. (2019). Roles of Inflammation, Neurogenic Inflammation, and Neuroinflammation in Pain. J. Anesth..

[121] Dantzer R., O’Connor J.C., Freund G.G., Johnson R.W., Kelley K.W. (2008). From Inflammation to Sickness and Depression: When the Immune System Subjugates the Brain. Nat. Rev. Neurosci..

[122] Steiner J., Bielau H., Brisch R., Danos P., Ullrich O., Mawrin C., Bernstein H.-G., Bogerts B. (2008). Immunological Aspects in the Neurobiology of Suicide: Elevated Microglial Density in Schizophrenia and Depression Is Associated with Suicide. J. Psychiatr. Res..

[123] Rock R.B., Gekker G., Hu S., Sheng W.S., Cheeran M., Lokensgard J.R., Peterson P.K. (2004). Role of Microglia in Central Nervous System Infections. Clin. Microbiol. Rev..

[124] Attwells S., Setiawan E., Wilson A.A., Rusjan P.M., Miler L., Xu C., Hutton C., Husain M.I., Kish S., Vasdev N. (2020). Replicating Predictive Serum Correlates of Greater Translocator Protein Distribution Volume in Brain. Neuropsychopharmacology.

[125] Watanabe M., Nakatani E., Yoshikawa H., Kanno T., Nariai Y., Yoshino A., Vieth M., Kinoshita Y., Sekine J. (2017). Oral Soft Tissue Disorders Are Associated with Gastroesophageal Reflux Disease: Retrospective Study. BMC Gastroenterol..

[126] Ohmure H., Kanematsu-Hashimoto K., Nagayama K., Taguchi H., Ido A., Tominaga K., Arakawa T., Miyawaki S. (2016). Evaluation of a Proton Pump Inhibitor for Sleep Bruxism. J. Dent. Res..

[127] Mengatto C.M., da Silveira Dalberto C., Scheeren B., Silva de Barros S.G. (2013). Association between Sleep Bruxism and Gastroesophageal Reflux Disease. J. Prosthet. Dent..

[128] Li Y., Yu F., Niu L., Hu W., Long Y., Tay F., Chen J. (2018). Associations among Bruxism, Gastroesophageal Reflux Disease, and Tooth Wear. J. Clin. Med..

[129] Li Y., Yu F., Niu L., Long Y., Tay F.R., Chen J. (2018). Association between Bruxism and Symptomatic Gastroesophageal Reflux Disease: A Case-Control Study. J. Dent..

[130] Kinoshita Y., Ishimura N., Ishihara S. (2018). Advantages and Disadvantages of Long-Term Proton Pump Inhibitor Use. J. Neurogastroenterol. Motil..

[131] William J.H., Danziger J. (2016). Proton-Pump Inhibitor-Induced Hypomagnesemia: Current Research and Proposed Mechanisms. World J. Nephrol..

[132] Hoorn E.J., van der Hoek J., de Man R.A., Kuipers E.J., Bolwerk C., Zietse R. (2010). A Case Series of Proton Pump Inhibitor–Induced Hypomagnesemia. Am. J. Kidney Dis..

[133] Cheungpasitporn W., Thongprayoon C., Kittanamongkolchai W., Srivali N., Edmonds P.J., Ungprasert P., O’Corragain O.A., Korpaisarn S., Erickson S.B. (2015). Proton Pump Inhibitors Linked to Hypomagnesemia: A Systematic Review and Meta-Analysis of Observational Studies. Ren. Fail..

[134] Hartman B., Donnelly-VanderLoo M., Watson T., O’Connor C., Madill J. (2016). Proton-Pump Inhibitor Therapy and Vitamin B _12_ Status in an Inpatient Hospital Setting. Appl. Physiol. Nutr. Metab..

[135] Lam J.R., Schneider J.L., Zhao W., Corley D.A. (2013). Proton Pump Inhibitor and Histamine 2 Receptor Antagonist Use and Vitamin B _12_ Deficiency. JAMA.

[136] Hirschowitz B.I., Worthington J., Mohnen J. (2008). Vitamin B12 Deficiency in Hypersecretors during Long-Term Acid Suppression with Proton Pump Inhibitors. Aliment. Pharmacol. Ther..

[137] Kumar N. (2014). Neurologic Aspects of Cobalamin (B12) Deficiency. Handb. Clin. Neurol..

[138] Freedberg D.E., Lebwohl B., Abrams J.A. (2014). The Impact of Proton Pump Inhibitors on the Human Gastrointestinal Microbiome. Clin. Lab. Med..

[139] Shi Y.-C., Cai S.-T., Tian Y.-P., Zhao H.-J., Zhang Y.-B., Chen J., Ren R.-R., Luo X., Peng L.-H., Sun G. (2019). Effects of Proton Pump Inhibitors on the Gastrointestinal Microbiota in Gastroesophageal Reflux Disease. Genom. Proteom. Bioinform..

[140] Takagi T., Naito Y., Inoue R., Kashiwagi S., Uchiyama K., Mizushima K., Tsuchiya S., Okayama T., Dohi O., Yoshida N. (2018). The Influence of Long-Term Use of Proton Pump Inhibitors on the Gut Microbiota: An Age-Sex-Matched Case-Control Study. J. Clin. Biochem. Nutr..

[141] Freedberg D.E., Toussaint N.C., Chen S.P., Ratner A.J., Whittier S., Wang T.C., Wang H.H., Abrams J.A. (2015). Proton Pump Inhibitors Alter Specific Taxa in the Human Gastrointestinal Microbiome: A Crossover Trial. Gastroenterology.

[142] Tsuda A., Suda W., Morita H., Takanashi K., Takagi A., Koga Y., Hattori M. (2015). Influence of Proton-Pump Inhibitors on the Luminal Microbiota in the Gastrointestinal Tract. Clin. Transl. Gastroenterol..

[143] Lucas López R., Grande Burgos M.J., Gálvez A., Pérez Pulido R. (2017). The Human Gastrointestinal Tract and Oral Microbiota in Inflammatory Bowel Disease: A State of the Science Review. APMIS.

[144] Fourie N.H., Wang D., Abey S.K., Sherwin L.B., Joseph P.V., Rahim-Williams B., Ferguson E.G., Henderson W.A. (2016). The Microbiome of the Oral Mucosa in Irritable Bowel Syndrome. Gut Microbes.

[145] Lo W., Chan W.W. (2013). Proton Pump Inhibitor Use and the Risk of Small Intestinal Bacterial Overgrowth: A Meta-Analysis. Clin. Gastroenterol. Hepatol..

[146] Spiegel B.M.R., Chey W.D., Chang L. (2008). Bacterial Overgrowth and Irritable Bowel Syndrome: Unifying Hypothesis or a Spurious Consequence of Proton Pump Inhibitors?. Am. J. Gastroenterol..

[147] Fujimori S. (2015). What Are the Effects of Proton Pump Inhibitors on the Small Intestine?. World J. Gastroenterol..

[148] Cheung K.S., Chan E.W., Wong A.Y.S., Chen L., Wong I.C.K., Leung W.K. (2018). Long-Term Proton Pump Inhibitors and Risk of Gastric Cancer Development after Treatment for *Helicobacter Pylori*: A Population-Based Study. Gut.

[149] Brusselaers N., Wahlin K., Engstrand L., Lagergren J. (2017). Maintenance Therapy with Proton Pump Inhibitors and Risk of Gastric Cancer: A Nationwide Population-Based Cohort Study in Sweden. BMJ Open.

[150] Ahn J.S. (2013). Acid Suppressive Drugs and Gastric Cancer: A Meta-Analysis of Observational Studies. World J. Gastroenterol..

[151] Ahn J., Chen C.Y., Hayes R.B. (2012). Oral Microbiome and Oral and Gastrointestinal Cancer Risk. Cancer Causes Control.

[152] Reiter R.J. (2002). Antioxidative Capacity of Melatonin. Handbook of Antioxidants.

[153] Konturek S.J., Zayachkivska O., Havryluk X.O., Brzozowski T., Sliwowski Z., Pawlik M., Konturek P.C., Cześnikiewicz-Guzik M., Gzhegotsky M.R., Pawlik W.W. (2007). Protective Influence of Melatonin against Acute Esophageal Lesions Involves Prostaglandins, Nitric Oxide and Sensory Nerves. J. Physiol. Pharm..

[154] Pereira R.d.S. (2006). Regression of Gastroesophageal Reflux Disease Symptoms Using Dietary Supplementation with Melatonin, Vitamins and Aminoacids: Comparison with Omeprazole. J. Pineal Res..

[155] Werbach M.R. (2008). Melatonin for the Treatment of Gastroesophageal Reflux Disease. Altern. Ther. Health Med..

[156] Hsiao E.Y., McBride S.W., Hsien S., Sharon G., Hyde E.R., McCue T., Codelli J.A., Chow J., Reisman S.E., Petrosino J.F. (2013). Microbiota Modulate Behavioral and Physiological Abnormalities Associated with Neurodevelopmental Disorders. Cell.

[157] Desbonnet L., Garrett L., Clarke G., Kiely B., Cryan J.F., Dinan T.G. (2010). Effects of the Probiotic Bifidobacterium Infantis in the Maternal Separation Model of Depression. Neuroscience.

[158] Liang S., Wang T., Hu X., Luo J., Li W., Wu X., Duan Y., Jin F. (2015). Administration of Lactobacillus Helveticus NS8 Improves Behavioral, Cognitive, and Biochemical Aberrations Caused by Chronic Restraint Stress. Neuroscience.

[159] Messaoudi M., Lalonde R., Violle N., Javelot H., Desor D., Nejdi A., Bisson J.-F., Rougeot C., Pichelin M., Cazaubiel M. (2011). Assessment of Psychotropic-like Properties of a Probiotic Formulation (*Lactobacillus Helveticus* R0052 and *Bifidobacterium Longum* R0175) in Rats and Human Subjects. Br. J. Nutr..

[160] Rao A.V., Bested A.C., Beaulne T.M., Katzman M.A., Iorio C., Berardi J.M., Logan A.C. (2009). A Randomized, Double-Blind, Placebo-Controlled Pilot Study of a Probiotic in Emotional Symptoms of Chronic Fatigue Syndrome. Gut Pathog..

[161] Breit S., Kupferberg A., Rogler G., Hasler G. (2018). Vagus Nerve as Modulator of the Brain–Gut Axis in Psychiatric and Inflammatory Disorders. Front. Psychiatry.

[162] Desbonnet L., Garrett L., Clarke G., Bienenstock J., Dinan T.G. (2008). The Probiotic Bifidobacteria Infantis: An Assessment of Potential Antidepressant Properties in the Rat. J. Psychiatr. Res..

[163] Auteri M., Zizzo M.G., Serio R. (2015). GABA and GABA Receptors in the Gastrointestinal Tract: From Motility to Inflammation. Pharmacol. Res..

[164] Baganz N.L., Blakely R.D. (2013). A Dialogue between the Immune System and Brain, Spoken in the Language of Serotonin. ACS Chem. Neurosci..

[165] Bravo J.A., Forsythe P., Chew M.V., Escaravage E., Savignac H.M., Dinan T.G., Bienenstock J., Cryan J.F. (2011). Ingestion of *Lactobacillus* Strain Regulates Emotional Behavior and Central GABA Receptor Expression in a Mouse via the Vagus Nerve. Proc. Natl. Acad. Sci. USA.

[166] Inoue T., Tanaka M., Masuda S., Ohue-Kitano R., Yamakage H., Muranaka K., Wada H., Kusakabe T., Shimatsu A., Hasegawa K. (2017). Omega-3 Polyunsaturated Fatty Acids Suppress the Inflammatory Responses of Lipopolysaccharide-Stimulated Mouse Microglia by Activating SIRT1 Pathways. Biochim. Biophys. Acta (BBA) Mol. Cell Biol. Lipids.

[167] Watson H., Mitra S., Croden F.C., Taylor M., Wood H.M., Perry S.L., Spencer J.A., Quirke P., Toogood G.J., Lawton C.L. (2018). A Randomised Trial of the Effect of Omega-3 Polyunsaturated Fatty Acid Supplements on the Human Intestinal Microbiota. Gut.

[168] Costantini L., Molinari R., Farinon B., Merendino N. (2017). Impact of Omega-3 Fatty Acids on the Gut Microbiota. Int. J. Mol. Sci..

[169] Liao Y., Xie B., Zhang H., He Q., Guo L., Subramanieapillai M., Fan B., Lu C., McIntyre R.S. (2019). Efficacy of Omega-3 PUFAs in Depression: A Meta-Analysis. Transl. Psychiatry.

[170] Ko G.D., Nowacki N.B., Arseneau L., Eitel M., Hum A. (2010). Omega-3 Fatty Acids for Neuropathic Pain. Clin. J. Pain.

[171] Galán-Arriero I., Serrano-Muñoz D., Gómez-Soriano J., Goicoechea C., Taylor J., Velasco A., Ávila-Martín G. (2017). The Role of Omega-3 and Omega-9 Fatty Acids for the Treatment of Neuropathic Pain after Neurotrauma. Biochim. Biophys. Acta (BBA) Biomembr..

[172] Goldberg R.J., Katz J. (2007). A Meta-Analysis of the Analgesic Effects of Omega-3 Polyunsaturated Fatty Acid Supplementation for Inflammatory Joint Pain. Pain.

[173] Frémont L. (2000). Biological Effects of Resveratrol. Life Sci..

[174] Alrafas H.R., Busbee P.B., Nagarkatti M., Nagarkatti P.S. (2019). Resveratrol Modulates the Gut Microbiota to Prevent Murine Colitis Development through Induction of Tregs and Suppression of Th17 Cells. J. Leukoc. Biol..

[175] Ma Y., Liu S., Shu H., Crawford J., Xing Y., Tao F. (2020). Resveratrol Alleviates Temporomandibular Joint Inflammatory Pain by Recovering Disturbed Gut Microbiota. Brain Behav. Immun..

[176] Koh A., de Vadder F., Kovatcheva-Datchary P., Bäckhed F. (2016). From Dietary Fiber to Host Physiology: Short-Chain Fatty Acids as Key Bacterial Metabolites. Cell.

[177] Banasiewicz T., Krokowicz Ł., Stojcev Z., Kaczmarek B.F., Kaczmarek E., Maik J., Marciniak R., Krokowicz P., Walkowiak J., Drews M. (2013). Microencapsulated Sodium Butyrate Reduces the Frequency of Abdominal Pain in Patients with Irritable Bowel Syndrome. Colorectal Dis..

[178] Huda-Faujan N., Abdulamir A.S., Fatimah A.B., Anas O.M., Shuhaimi M., Yazid A.M., Loong Y.Y. (2010). The Impact of the Level of the Intestinal Short Chain Fatty Acids in Inflammatory Bowel Disease Patients Versus Healthy Subjects. Open Biochem. J..

[179] Kukkar A., Singh N., Jaggi A.S. (2014). Attenuation of Neuropathic Pain by Sodium Butyrate in an Experimental Model of Chronic Constriction Injury in Rats. J. Formos. Med. Assoc..

[180] Vinolo M.A.R., Rodrigues H.G., Nachbar R.T., Curi R. (2011). Regulation of Inflammation by Short Chain Fatty Acids. Nutrients.

[181] Hardeland R. (2018). Melatonin and Inflammation-Story of a Double-Edged Blade. J. Pineal Res..

[182] Chen C.-Q. (2011). Distribution, Function and Physiological Role of Melatonin in the Lower Gut. World J. Gastroenterol..

[183] Huether G. (1994). Melatonin Synthesis in the Gastrointestinal Tract and the Impact of Nutritional Factors on Circulating Melatonin. Ann. N. Y. Acad. Sci.

[184] Grigorov I., Bogojević D., Jovanović S., Petrović A., Ivanović-Matić S., Zolotarevski L., Poznanović G., Martinović V. (2014). Hepatoprotective Effects of Melatonin against Pronecrotic Cellular Events in Streptozotocin-Induced Diabetic Rats. J. Physiol. Biochem..

[185] Welin A.-K., Svedin P., Lapatto R., Sultan B., Hagberg H., Gressens P., Kjellmer I., Mallard C. (2007). Melatonin Reduces Inflammation and Cell Death in White Matter in the Mid-Gestation Fetal Sheep Following Umbilical Cord Occlusion. Pediatric Res..

[186] Ohashi N., Ishigaki S., Isobe S. (2019). The Pivotal Role of Melatonin in Ameliorating Chronic Kidney Disease by Suppression of the Renin–Angiotensin System in the Kidney. Hypertens. Res..

[187] Brennan R., Jan J.E., Lyons C.J. (2007). Light, Dark, and Melatonin: Emerging Evidence for the Importance of Melatonin in Ocular Physiology. Eye.

[188] Tarocco A., Caroccia N., Morciano G., Wieckowski M.R., Ancora G., Garani G., Pinton P. (2019). Melatonin as a Master Regulator of Cell Death and Inflammation: Molecular Mechanisms and Clinical Implications for Newborn Care. Cell Death Dis..

[189] Ma N., Zhang J., Reiter R.J., Ma X. (2020). Melatonin Mediates Mucosal Immune Cells, Microbial Metabolism, and Rhythm Crosstalk: A Therapeutic Target to Reduce Intestinal Inflammation. Med. Res. Rev..

[190] Huang H., Wang Z., Weng S.-J., Sun X.-H., Yang X.-L. (2013). Neuromodulatory Role of Melatonin in Retinal Information Processing. Prog. Retin. Eye Res..

[191] Chen X., Eslamfam S., Fang L., Qiao S., Ma X. (2017). Maintenance of Gastrointestinal Glucose Homeostasis by the Gut-Brain Axis. Curr. Protein Pept. Sci..

[192] Wilhelmsen M., Amirian I., Reiter R.J., Rosenberg J., Gögenur I. (2011). Analgesic Effects of Melatonin: A Review of Current Evidence from Experimental and Clinical Studies. J. Pineal Res..

[193] Danilov A., Kurganova J. (2016). Melatonin in Chronic Pain Syndromes. Pain Ther..

[194] Hardeland R. (2012). Melatonin in Aging and Disease -Multiple Consequences of Reduced Secretion, Options and Limits of Treatment. Aging Dis..

[195] Ambriz-Tututi M., Rocha-González H.I., Cruz S.L., Granados-Soto V. (2009). Melatonin: A Hormone That Modulates Pain. Life Sci..

[196] Esposito E., Paterniti I., Mazzon E., Bramanti P., Cuzzocrea S. (2010). Melatonin Reduces Hyperalgesia Associated with Inflammation. J. Pineal Res..

[197] Acuna-Castroviejo D., Escames G., Reiter R.J. (2006). Melatonin Therapy in Fibromyalgia. J. Pineal Res..

[198] Citera G., Arias M.A., Maldonado-Cocco J.A., La´zaro M.A., Rosemffet M.G., Brusco L.I., Scheines E.J., Cardinalli D.P. (2000). The Effect of Melatonin in Patients with Fibromyalgia: A Pilot Study. Clin. Rheumatol..

[199] Srinivasan V., Lauterbach E.C., Yu Ho K., Acuna-Castroviejo D., Zakaria R., Brzezinski A. (2012). Melatonin in Antinociception: Its Therapeutic Applications. Curr. Neuropharmacol..

[200] Zhu C., Xu Y., Duan Y., Li W., Zhang L., Huang Y., Zhao W., Wang Y., Li J., Feng T. (2017). Exogenous Melatonin in the Treatment of Pain: A Systematic Review and Meta-Analysis. Oncotarget.

[201] Myung S.-K. (2020). Analgesic Efficacy of Melatonin: A Meta-Analysis of Randomized, Double-Blind, Placebo-Controlled Trials. J. Clin. Med..

[202] Xie S., Fan W., He H., Huang F. (2020). Role of Melatonin in the Regulation of Pain. J. Pain Res..

[203] Huang C.-T., Chiang R.P.-Y., Chen C.-L., Tsai Y.-J. (2014). Sleep Deprivation Aggravates Median Nerve Injury-Induced Neuropathic Pain and Enhances Microglial Activation by Suppressing Melatonin Secretion. Sleep.

[204] Caumo W., Hidalgo M.P., Souza A., Torres I.L.d.S., Conceicao Antunes L. (2019). Melatonin Is a Biomarker of Circadian Dysregulation and Is Correlated with Major Depression and Fibromyalgia Symptom Severity. J. Pain Res..

[205] Vidor L.P., Torres I.L.S., Custódio de Souza I.C., Fregni F., Caumo W. (2013). Analgesic and Sedative Effects of Melatonin in Temporomandibular Disorders: A Double-Blind, Randomized, Parallel-Group, Placebo-Controlled Study. J. Pain Symptom Manag..

[206] Erden S. (2020). Sleep-Related Bruxism Response to Melatonin Treatment. J. Child Adolesc. Psychopharmacol..

[207] Zarezadeh M., Khorshidi M., Emami M., Janmohammadi P., Kord-varkaneh H., Mousavi S.M., Mohammed S.H., Saedisomeolia A., Alizadeh S. (2020). Melatonin Supplementation and Pro-Inflammatory Mediators: A Systematic Review and Meta-Analysis of Clinical Trials. Eur. J. Nutr..

[208] MacInnis M.J., Dziedzic C.E., Wood E., Oikawa S.Y., Phillips S.M. (2020). Presleep α-Lactalbumin Consumption Does Not Improve Sleep Quality or Time-Trial Performance in Cyclists. Int. J. Sport Nutr. Exerc. Metab..

[209] Besag F.M.C., Vasey M.J., Lao K.S.J., Wong I.C.K. (2019). Adverse Events Associated with Melatonin for the Treatment of Primary or Secondary Sleep Disorders: A Systematic Review. CNS Drugs.

[210] Seabra M.d.L.v., Bignotto M., Pinto L.R., Tufik S. (2000). Randomized, Double-Blind Clinical Trial, Controlled with Placebo, of the Toxicology of Chronic Melatonin Treatment. J. Pineal Res..

[211] Andersen L.P.H., Gögenur I., Rosenberg J., Reiter R.J. (2016). The Safety of Melatonin in Humans. Clin. Drug Investig..

[212] Rajaratnam S.M.W., Dijk D.-J., Middleton B., Stone B.M., Arendt J. (2003). Melatonin Phase-Shifts Human Circadian Rhythms with No Evidence of Changes in the Duration of Endogenous Melatonin Secretion or the 24-Hour Production of Reproductive Hormones. J. Clin. Endocrinol. Metab..

[213] Luboshitzky R., Levi M., Shen-Orr Z., Blumenfeld Z., Herer P., Lavie P. (2000). Long-Term Melatonin Administration Does Not Alter Pituitary-Gonadal Hormone Secretion in Normal Men. Hum. Reprod..

[214] Voordouw B.C., Euser R., Verdonk R.E., Alberda B.T., de Jong F.H., Drogendijk A.C., Fauser B.C., Cohen M. (1992). Melatonin and Melatonin-Progestin Combinations Alter Pituitary-Ovarian Function in Women and Can Inhibit Ovulation. J. Clin. Endocrinol. Metab..

[215] Aleem F.A., Weitzman E.D., Weinberg U. (1984). Suppression of Basal Luteinizing Hormone Concentrations by Melatonin in Postmenopausal Women. Fertil. Steril..

[216] Cipolla-Neto J., Amaral F.G., Soares J.J.M., Gallo C.C., Furtado A., Cavaco J.E., Gonçalves I., Santos C.R.A., Quintela T. (2022). The Crosstalk between Melatonin and Sex Steroid Hormones. Neuroendocrinology.

[217] Gonzalez A., Cos S., Martinez-Campa C., Alonso-Gonzalez C., Sanchez-Mateos S., Mediavilla M.D., Sanchez-Barcelo E.J. (2008). Selective Estrogen Enzyme Modulator Actions of Melatonin in Human Breast Cancer Cells. J. Pineal Res..

[218] Cos S., Gonzalez A., Martinez-Campa C., Mediavilla M., Alonso-Gonzalez C., Sanchez-Barcelo E. (2008). Melatonin as a Selective Estrogen Enzyme Modulator. Curr. Cancer Drug Targets.

[219] Greene C.S., Manfredini D. (2021). Transitioning to Chronic Temporomandibular Disorder Pain: A Combination of Patient Vulnerabilities and Iatrogenesis. J. Oral Rehabil..

[220] Kindler S., Schwahn C., Bernhardt O., Söhnel A., Mksoud M., Biffar R., Meyer G., Völzke H., Metelmann H., Grabe H. (2019). Association Between Symptoms of Posttraumatic Stress Disorder and Signs of Temporomandibular Disorders in the General Population. J. Oral Facial Pain Headache.

[221] Najavits L.M., Weiss R.D., Shaw S.R. (1997). The Link between Substance Abuse and Posttraumatic Stress Disorder in Women. A Research Review. Am. J. Addict..

[222] Pietrzak R.H., Goldstein R.B., Southwick S.M., Grant B.F. (2011). Prevalence and Axis I Comorbidity of Full and Partial Posttraumatic Stress Disorder in the United States: Results from Wave 2 of the National Epidemiologic Survey on Alcohol and Related Conditions. J. Anxiety Disord..

[223] Salk R.H., Hyde J.S., Abramson L.Y. (2017). Gender Differences in Depression in Representative National Samples: Meta-Analyses of Diagnoses and Symptoms. Psychol. Bull..

[224] Prather A.A., Carroll J.E., Fury J.M., McDade K.K., Ross D., Marsland A.L. (2009). Gender Differences in Stimulated Cytokine Production Following Acute Psychological Stress. Brain Behav. Immun..

[225] Palma-Gudiel H., Peralta V., Deuschle M., Navarro V., Fañanás L. (2019). Epigenetics-by-Sex Interaction for Somatization Conferred by Methylation at the Promoter Region of SLC6A4 Gene. Prog. Neuro-Psychopharmacol. Biol. Psychiatry.

[226] Baumeister D., Akhtar R., Ciufolini S., Pariante C.M., Mondelli V. (2016). Childhood Trauma and Adulthood Inflammation: A Meta-Analysis of Peripheral C-Reactive Protein, Interleukin-6 and Tumour Necrosis Factor-α. Mol. Psychiatry.

[227] Zhang B., Wing Y.-K. (2006). Sex Differences in Insomnia: A Meta-Analysis. Sleep.

[228] Irwin M.R. (2006). Sleep Deprivation and Activation of Morning Levels of Cellular and Genomic Markers of Inflammation. Arch. Intern. Med..

[229] Irwin M.R., Wang M., Ribeiro D., Cho H.J., Olmstead R., Breen E.C., Martinez-Maza O., Cole S. (2008). Sleep Loss Activates Cellular Inflammatory Signaling. Biol. Psychiatry.

[230] Roane B.M., Taylor D.J. (2008). Adolescent Insomnia as a Risk Factor for Early Adult Depression and Substance Abuse. Sleep.

[231] Wong M.M., Brower K.J., Zucker R.A. (2009). Childhood Sleep Problems, Early Onset of Substance Use and Behavioral Problems in Adolescence. Sleep Med..

[232] Telzer E.H., Fuligni A.J., Lieberman M.D., Galván A. (2013). The Effects of Poor Quality Sleep on Brain Function and Risk Taking in Adolescence. Neuroimage.

[233] Morgan P.T., Pace-Schott E.F., Sahul Z.H., Coric V., Stickgold R., Malison R.T. (2006). Sleep, Sleep-Dependent Procedural Learning and Vigilance in Chronic Cocaine Users: Evidence for Occult Insomnia. Drug Alcohol Depend..

[234] Debell F., Fear N.T., Head M., Batt-Rawden S., Greenberg N., Wessely S., Goodwin L. (2014). A Systematic Review of the Comorbidity between PTSD and Alcohol Misuse. Soc. Psychiatry Psychiatr. Epidemiol..

[235] Colrain I.M., Nicholas C.L., Baker F.C. (2014). Alcohol and the Sleeping Brain. Handb. Clin. Neurol..

[236] Scrima L., Broudy M., Nay K.N., Cohn M.A. (1982). Increased Severity of Obstructive Sleep Apnea After Bedtime Alcohol Ingestion: Diagnostic Potential and Proposed Mechanism of Action. Sleep.

[237] Roehrs T. (1999). Ethanol as a Hypnotic in Insomniacs Self Administration and Effects on Sleep and Mood. Neuropsychopharmacology.

[238] Wilson S., Argyropoulos S. (2005). Antidepressants and Sleep. Drugs.

[239] Benedict C., Brooks S.J., O’Daly O.G., Almèn M.S., Morell A., Åberg K., Gingnell M., Schultes B., Hallschmid M., Broman J.-E. (2012). Acute Sleep Deprivation Enhances the Brain’s Response to Hedonic Food Stimuli: An FMRI Study. J. Clin. Endocrinol. Metab..

[240] Kim Y.S., Kim N., Kim G.H. (2016). Sex and Gender Differences in Gastroesophageal Reflux Disease. J. Neurogastroenterol. Motil..

[241] Lee S.P., Sung I.-K., Kim J.H., Lee S.-Y., Park H.S., Shim C.S. (2015). The Effect of Emotional Stress and Depression on the Prevalence of Digestive Diseases. J. Neurogastroenterol. Motil..

[242] On Z.X., Grant J., Shi Z., Taylor A.W., Wittert G.A., Tully P.J., Hayley A.C., Martin S. (2017). The Association between Gastroesophageal Reflux Disease with Sleep Quality, Depression, and Anxiety in a Cohort Study of Australian Men. J. Gastroenterol. Hepatol..

[243] You Z.-H., Perng C.-L., Hu L.-Y., Lu T., Chen P.-M., Yang A.C., Tsai S.-J., Huang Y.-S., Chen H.-J. (2015). Risk of Psychiatric Disorders Following Gastroesophageal Reflux Disease: A Nationwide Population-Based Cohort Study. Eur. J. Intern. Med..

[244] Baker L.H., Lieberman D., Oehlke M. (1995). Psychological Distress in Patients with Gastroesophageal Reflux Disease. Am. J. Gastroenterol..

[245] Fillingim R.B., Ohrbach R., Greenspan J.D., Knott C., Diatchenko L., Dubner R., Bair E., Baraian C., Mack N., Slade G.D. (2013). Psychological Factors Associated with Development of TMD: The OPPERA Prospective Cohort Study. J. Pain.

[246] Jaggar M., Rea K., Spichak S., Dinan T.G., Cryan J.F. (2020). You’ve Got Male: Sex and the Microbiota-Gut-Brain Axis across the Lifespan. Front. Neuroendocrinol..

[247] d’Hennezel E., Abubucker S., Murphy L.O., Cullen T.W. (2017). Total Lipopolysaccharide from the Human Gut Microbiome Silences Toll-Like Receptor Signaling. mSystems.

[248] Santos-Marcos J.A., Rangel-Zuñiga O.A., Jimenez-Lucena R., Quintana-Navarro G.M., Garcia-Carpintero S., Malagon M.M., Landa B.B., Tena-Sempere M., Perez-Martinez P., Lopez-Miranda J. (2018). Influence of Gender and Menopausal Status on Gut Microbiota. Maturitas.

[249] Sinha T., Vich Vila A., Garmaeva S., Jankipersadsing S.A., Imhann F., Collij V., Bonder M.J., Jiang X., Gurry T., Alm E.J. (2019). Analysis of 1135 Gut Metagenomes Identifies Sex-Specific Resistome Profiles. Gut Microbes.

[250] Mohlin B., Axelsson S., Paulin G., Pietilä T., Bondemark L., Brattström V., Hansen K., Holm A.-K. (2007). TMD in Relation to Malocclusion and Orthodontic Treatment. Angle Orthod..

[251] Bueno C.H., Pereira D.D., Pattussi M.P., Grossi P.K., Grossi M.L. (2018). Gender Differences in Temporomandibular Disorders in Adult Populational Studies: A Systematic Review and Meta-Analysis. J. Oral Rehabil..

